# Boron-neutron Capture on Activated Carbon for Hydrogen Storage

**DOI:** 10.1038/s41598-019-39417-6

**Published:** 2019-02-27

**Authors:** Jimmy Romanos, Matthew Beckner, Matthew Prosniewski, Tyler Rash, Mark Lee, J. David Robertson, Lucyna Firlej, Bogdan Kuchta, Peter Pfeifer

**Affiliations:** 1 Department of Natural Sciences, Lebanese American University, Byblos, P.O.Box 36, Lebanon; 2 Department of Physics, University of Missouri, Columbia, 65201 Missouri United States; 3 Department of Chemistry, University of Missouri, Columbia, 65201 Missouri United States; 4 MU Research Reactor (MURR), University of Missouri, Columbia, 65201 Missouri United States; 5 Laboratoire Charles Coulomb, UMR5221, University of Montpellier - CNRS, Montpellier, France; 6 Laboratoire MARIDEL, CNRS, Aix-Marseille University, Marseille, France

## Abstract

This work investigates the effects of neutron irradiation on nitrogen and hydrogen adsorption in boron-doped activated carbon. Boron-neutron capture generates an energetic lithium nucleus, helium nucleus, and gamma photons, which can alter the surface and structure of pores in activated carbon. The defects introduced by fission tracks are modeled assuming the slit-shaped pores geometry. Sub-critical nitrogen adsorption shows that nitrogen molecules cannot probe the defects created by fission tracks. Hydrogen adsorption isotherms of irradiated samples indicate higher binding energies compared to their non-irradiated parent samples.

## Introduction

Hydrogen is currently stored and transported in compressed or liquefied form. Hydrogen storage by chemisorption or physisorption in host materials emerged as an alternative, practical and safe storage technology for the light-duty vehicles. In metal and chemical hydrides, hydrogen is linked to the host through covalent, ionic, or metallic-type bonds. Therefore, chemisorption is hardly reversible and hydrogen is only released at high temperature or upon exposure to a catalyst^1,2^. In physisorption, hydrogen molecules are adsorbed on the sorbent surface by weak van der Waals forces as a high density fluid^3–8^. In consequence the heat of adsorption of hydrogen is relatively small, resulting in low storage capacities.

Despite tremendous research efforts to develop an efficient hydrogen sorbent, today no ma- terial meets the storage targets set by the US Department of Energy (DOE)^9^. The most pertinent parameters for development of hydrogen sorbent are gravimetric and volumetric storage capacities of the material. The targets to be reached by 2020, 2025, and in the ultimate limit are respectively set to 0.045, 0.055, and 0.065 kg H_2_/kg (for gravimetric storage), and 0.030, 0.040, and 0.050 kg H_2_/L (for volumetric storage). It is important to note that these targets are defined for a complete storage system (counting the mass and volume occupied by the valves, regulators, pipes, materials, and other engineering components), and not for the sorbent material only. The most promising and widely investigated materials for hydrogen storage by physisorption are metal-organic framework (MOFs) and activated carbons (ACs).

ACs are considered as effective hydrogen adsorbents primarily due to their high porosity and large surface areas. Several computational models have defined the optimal pore structure and sur- face chemistry for ACs for hydrogen physisorption^10–12^. Many experimental attempts to improve the surface chemistry and pore size distribution of the existing ACs were reported in the literature. The pore structure were engineered by optimizing the activation process (by selecting an optimal activation temperature and activation agent concentration^13,14^). Surface chemistry was modified by doping the carbon lattice with boron, aluminum, lithium, calcium and other elements^15,16^. However, all these attempts led to a marginal improvement of experimentally measured hydrogen storage capacities, in agreement with recent discussion of the theoretical limit of existing AC structure for hydrogen storage^17^.

In this work, we present a new approach of ACs structure modification by high energetic fission tracks. For that, boron doped ACs were irradiated by neutrons in order to create defects, and modify the pore surface and structure. The effects of neutron irradiation of carbon based materials was first studied by Spalaris *et al*.^18^. The authors showed that neutron irradiation of graphite causes a decrease of samples’ surface area. They suggested that upon irradiation the graphite crystallites expend to occupy voids in their immediate vicinity; in consequence the observed sample microporosity decreased. Additional study of boron neutron capture in graphite were reported by Cadenhead and Chung^19,20^. It has been also shown that the amount of moisture adsorbed in neutron irradiated ACs and silica increased respectively by 18% and 23% (with respect to non- irradiated samples), although only a moderate (<100 m^2^/g) increase of samples surface area has been observed^21,22^. Previous attempts of carbon irradiation by neutrons were performed for low surface area carbons. Here we present the first attempt to modify the pore structure of boron- doped high-surface area carbon (3300 m^2^/g), and report a relevant change in hydrogen adsorption isotherm after neutron irradiation.

### Modeling of high-binding-energy sites created by fission tracks

The isotopic abundance of ^10^B is around 20%. This light element shows a strong tendency to bind with thermal neutrons and form an excited ^11^B nucleus. This nucleus is unstable, and decays via fission, producing a lithium nucleus, helium nucleus, and gamma photon.

$${}_{5}{}^{10}B+\,{}_{0}{}^{1}{\rm{n}}\to \,{[{}_{5}{}^{11}{\rm{B}}]}^{\ast }\to \,{}_{2}{}^{4}{\rm{H}}{\rm{e}}+{}_{3}{}^{7}{\rm{L}}{\rm{i}}+{\rm{2.79}}\,{\rm{Mev}}\,(6 \% )\,$$

$${}_{5}{}^{10}{\rm{B}}+\,{}_{0}{}^{1}{\rm{n}}\to \,{[{}_{5}{}^{11}B]}^{\ast }\to \,{}_{2}{}^{4}{\rm{H}}{\rm{e}}+{}_{3}{}^{7}{\rm{L}}{\rm{i}}+2.31\,{\rm{Mev}}+{\rm{\gamma }}\,(94 \% )$$

The rate of the reaction R (number of nuclear reactions occurring per second) depends on the boron content of the sample N, the thermal (*φ*_*th*_)and epithermal (*φ*_*epi*_) fluxes of neutrons, and their cross sections (*σ*_*th*_ and *σ*_*epi*_, respectively) It is given by the following formula:

1 $$R={R}_{th}+{R}_{epi}=({\varphi }_{th}\cdot {\sigma }_{th}+{\varphi }_{epi}\cdot {\sigma }_{epi})N$$

We used the neutron beam thermal flux *ϕ*_*th*_ = 8 *·* 10^13^ *neutrons/cm*^2^ *· s*, and the epithermal flux *ϕ*_*epi*_ = 4.8 *·* 10^12^ *neutrons/cm*^2^.*s*. The corresponding cross sections are: *σ*_*th*_ = 3.84 *·* 10^*−*21^ *cm*^2^ (for thermal neutrons) and *σ*_*epi*_ = 1.73 *·* 10^*−*21^ *cm*^2^ (for epithermal neutrons). The number of boron atoms (N) in the sample can be estimated using the formula

2 $$N=\frac{m}{M}\cdot {N}_{A}\cdot B\cdot \eta $$

where *m* is the sample mass, *M* is the molar mass of ^10^B, *B* is the mass percent of boron in the sample, and *η* = 0.199 is the isotopic abundance of ^10^B.

**Figure 1:**
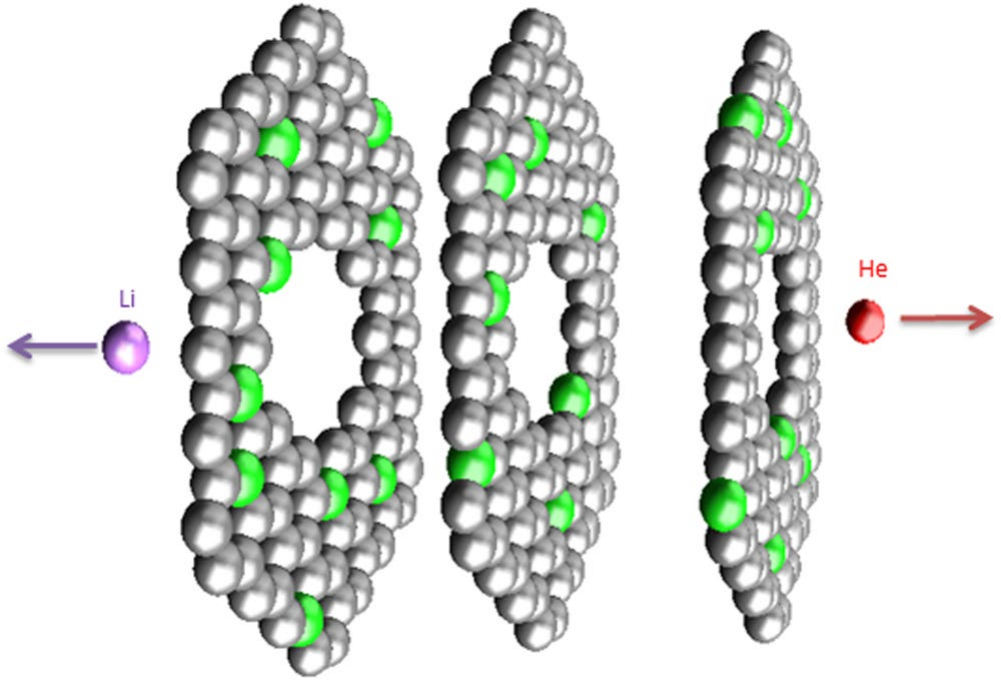
Defects introduced to slit-shaped, boron substituted carbon-based pore structure by fis- sion tracks from high energetic alpha and Li nuclei. The green spheres represent substitutional boron atoms.

The number of tracks created during sample irradiation is given by

3 $${N}_{tr}=2\cdot R\cdot {T}_{irr}$$

where *t*_*irr*_ is the irradiation time in seconds. The factor two in Eq. (3) takes into account the fact that every fission event produces two tracks, one by the emitted alpha particle and the other by the Li nucleus (Fig. 1). The time evolution of ^10^B amount is described by the equation:

4 $$N={N}_{0}{e}^{-({\varphi }_{th}\cdot {\sigma }_{th}+{\varphi }_{epi}\cdot {\sigma }_{epi}){T}_{irr}}$$

where *N*_0_ is the initial number of ^10^B and *N* is the number of ^10^B atoms remaining after the irradiation time *t*_*irr*_. This implies that after 52 minutes of irradiation, 0.1% of ^10^B atoms would have transformed, and after ~25.4 days of irradiation, half of them would have transformed.

Several techniques allows to study the microporous structure of ACs: gas adsorption, small angle x-ray scattering, high resolution transmission electron microscopy, and others^23–28^. All studies conclude that the structure of ACs consists of randomly oriented curved graphene fragments^29,30^. The defects created by fission tracks resulting from boron-neutron capture have been modeled assuming the widely used classical slit-shaped pores structure^31–36^. The distance L at which He and Li particles travel in ACs is estimated using the mean distance of helium penetration in carbon based materials^37^. The number of holes *n* created by *N*_*tr*_ tracks is approximated to be:

5 $$n={N}_{tr}\cdot \frac{L}{D+2{r}_{1}}$$

where *N*_*tr*_ is the number of tracks created by He and Li nuclei, *D* is the average pore width, and *r* is the distance between graphene pore wall and the first layer of adsorbed H_2_ which is approximately equal to 3.1 Å^38^. The carbon-to-carbon pore width (*D* + 2*r*) can be estimated from

6 $$D+2{r}_{1}=\frac{2}{{{\rm{\Sigma }}}_{i}\cdot {\rho }_{app}}$$

where Σ_*i*_ (*m*^2^*/g*) is the initial surface area before irradiation, *ρ*_*app*_ (*g/m*^3^) is the apparent sample density determined from subcritical nitrogen isotherms at 77 K.Substituting Eqs (3) and (6) into (5) leads to:

7 $$n=R\cdot {T}_{irr}\cdot L\cdot {{\rm{\Sigma }}}_{i}\cdot {\rho }_{app}$$

The surface area created (Σ_+_) and destroyed (Σ_*−*_) by fission tracks of width *w* are given by the following formulas:

7.a $${{\rm{\Sigma }}}_{+}=\,\frac{n}{M}\cdot {\pi }^{2}\cdot w\cdot r$$

7.b $${{\rm{\Sigma }}}_{-}=\,\frac{n}{M}\cdot \pi \cdot \frac{{w}^{2}}{2}$$

**Figure 2:**
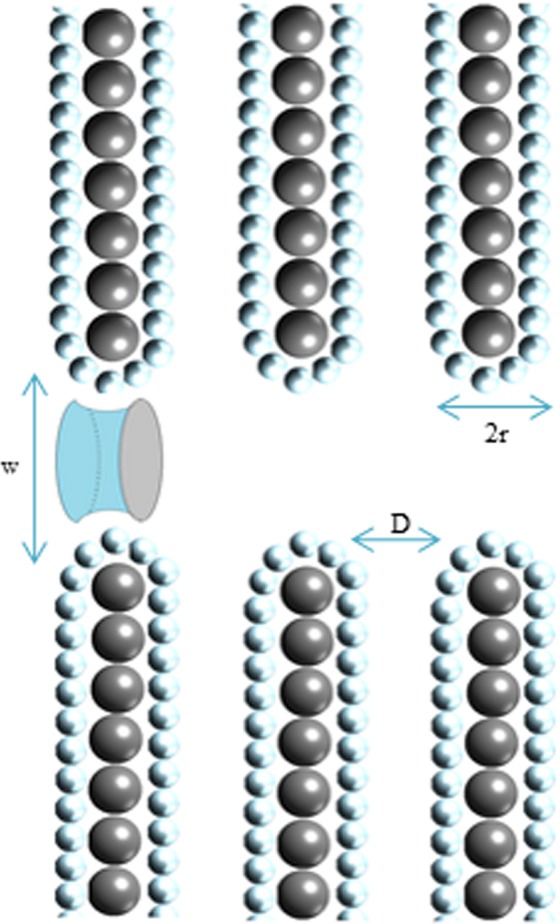
Lateral representation of fission tracks. Grey surface of the cylinder represents the pars of pore surface lost by irradiation. Blue part is the additional surface created by fission tracks.

Both surfaces are represented in Fig. 2. The overall change in specific surface area is given by the following equation:

8 $${\rm{\Delta }}{\rm{\Sigma }}={{\rm{\Sigma }}}_{+}-{{\rm{\Sigma }}}_{-}=\frac{R\cdot {T}_{irr}\cdot L\cdot {{\rm{\Sigma }}}_{i}\cdot {\rho }_{app}}{m}({\pi }^{2}\cdot w\cdot r-\pi \cdot \frac{{w}^{2}}{2})$$

Therefore, to obtain an additional surface area by neutron irradiation of activated carbon (ΔΣ > 0), *w* must be smaller than 2 *· π ·* r. In addition, the optimal pore width (*w*_*opt*_ = *π ·* r) should be 9.3 Å

## Experimental

### Sample preparation

AC samples were prepared in a multi-step process from corncob consisting of successive chemical activation by phosphoric acid and potassium hydroxide. During the first activation, corn- cob was soaked with phosphoric acid for 12 hours in an oven at 45 °C. The mixture was charred at 480 °C in a nitrogen environment. Charred carbon was then washed with hot water until neu- tralized (pH = 7). The second chemical activation with KOH solution was performed at 790 °C and KOH to carbon ratio of 3:1. The resulting material, after being washed with water (pH = 7) and dried, was then doped by vapor deposition of decaborane. For that, AC and B_10_H_14_ were first mixed and degassed for 1 hour at −30 °C under dynamic pumping, then the reaction cell was sealed under vacuum. The sealed cell was heated to 250 °C and maintained at this temperature for 4 hours, to allow B_10_H_14_ to sublimate, fill pore space, decompose, and form a sub-monolayer of B_10*x*_H_*z*_ on the pore walls. The sealed cell was then flushed with argon, cooled to 20 °C, transferred under an argon atmosphere to a high-pressure cell and sealed again. Finally, the sample was annealed at 600 °C to decompose the B_10_H_14_.

Boron doped samples were neutron irradiated for 1 minute at the University of Missouri Research Reactor (MURR). Boron content in the boron doped samples is determined by prompt- gamma neutron activation analysis (PGNAA). After irradiation, the samples were etched with hydrogen peroxide to force oxidation in order to create uniform sub-nm pores crisscrossing the pre-fission pore walls. 1 ml of 30% H_2_O_2_ and 70% H_2_O solution was added to the irradiated sample and heated to 750 °C for 1 hour. The sample was then placed in a vacuum oven at 600 °C and annealed for 15 hours. The boron content in the resulting sample is 1.4 wt %. The formation of B-C bonds was confirmed by Fourier transform infrared spectroscopy in our previous paper^12^.

### Subcritical nitrogen adsorption

Subcritical nitrogen isotherms at 77 K were obtained using an Autosorb-1C (Quantachrome Instruments). Specific surface areas (Σ) are determined from sub-critical nitrogen isotherms using Brunauer-Emett-Teller (BET) theory in the pressure range of 0.01–0.03 P/P_0_, suitable for analysis of microporous materials. Surface areas larger 1000 m^2^/g were rounded to the nearest hundred. The total pore volume (*V*_*tot*_) is measured at a relative pressure of 0.995 P/P_0_. The porosity (*φ*), defined as the fraction of sample volume occupied by open pores, is calculated as follow

9 $$\begin{array}{rcl}\varphi  & = & {[1+{({\rho }_{skel}\cdot \frac{{{\rm{V}}}_{tot}}{{m}_{s}})}^{-1}]}^{-1}\\ {\rho }_{app} & = & \,{\rho }_{skel}(1-\varphi )\end{array}$$

where *ρ*_*skel*_ is the skeletal density of the sample, assumed to be 2.0 g/cm^3^. Typical skeletal densities of amorphous carbons are between 1.8 and 2.1 g/cm^3^ ^38^.

Quenched solid-density functional theory (QSDFT)^35,36^ for infinite slit-shaped pores is used to calculate the pore-size distribution. QSDFT is a modified version of the non-local density functional theory (NLDFT). NLDFT which assumes a flat graphitic pore structure, has a significant drawback when applied to nanoporous AC, in which pore walls heterogeneities prevent layering transitions, thus leading to false minimums in the pore-size distribution. This artifact has been completely eliminated in QSDFT that takes into account surface roughness and heterogeneity.

### Supercritical hydrogen adsorption

Hydrogen (99.999% purity) adsorption isotherms were measured volumetrically using Hiden HTP-1 volumetric sorption analyzer. Hydrogen gravimetric excess adsorption isotherms were measured at T = 80 K and pressures ranging from 1 to 100 bar. Dry sample mass was determined after annealing the sample at 400 °C and dynamic vacuum (20 torr) for two hours.

## Results and Discussion

**Figure 3:**
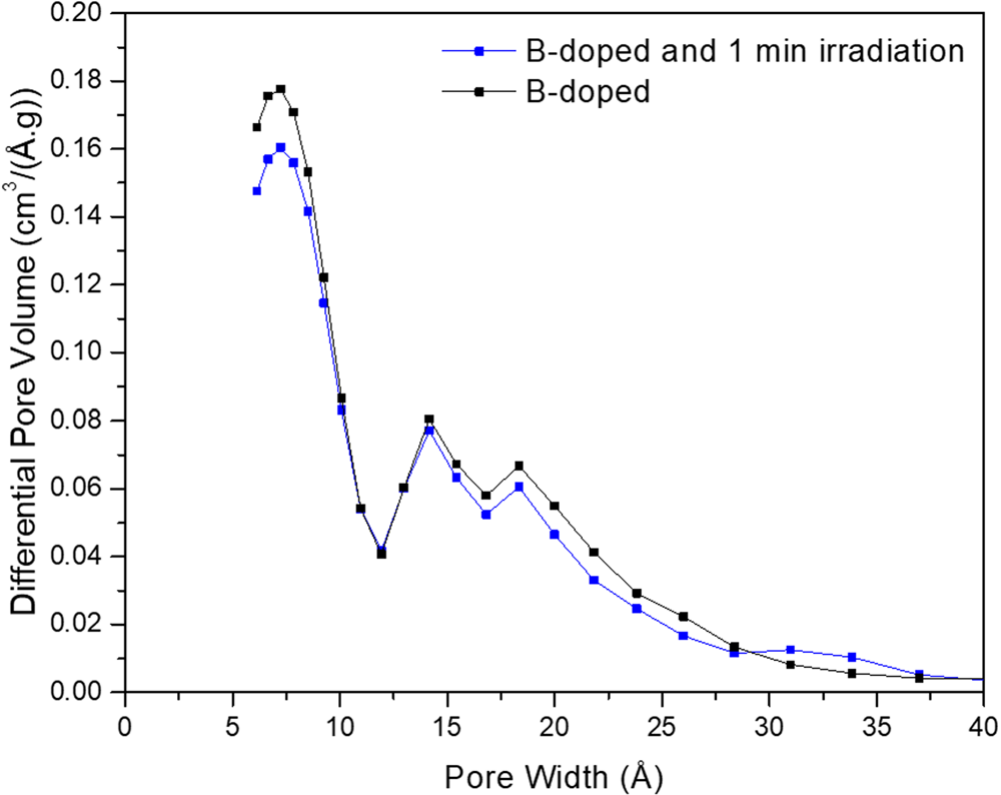
Pore size distribution for non-irradiated and 1 minute neutron irradiated B-doped sam- ples.

Nitrogen adsorption isotherms, BET surface area, porosity, and pore size distribution showed marginal change upon neutron irradiation. The samples specific surface was 3300 m^2^/g before, and 3100 m^2^/g after irradiation. This decrease remains within the estimated experimental 5% error. Similarly, the changes of porosity are small; 0.79 before and 0.78 after irradiation. The pore size distribution does not change upon neutron irradiation (Fig. 3). In both samples the pores width are smaller than 40 Å, with the main peak located at 7.5 Å. This observation is con- sistent with the physical picture of fission. The fission products displace isolated atoms and break bonds between neighboring atoms, but, no matter how energetic they are, they will never remove atoms from the solid or displace them in such a way that would create new regions of high and low carbon density. Such density modulation would be required to create detectable new pores or new surface area, that could be probed by nitrogen molecules. It is worth to recall that the N_2_ molecules are relatively large, and the width of the tracks and defects created during irradiation is small, approximately equal to 2 *· π ·* r. In consequence, there is no difference between nitrogen adsorption isotherms measured in irradiated and non-irradiated material. Obviously, the mass and skeletal density are also conserved during fission.

**Figure 4:**
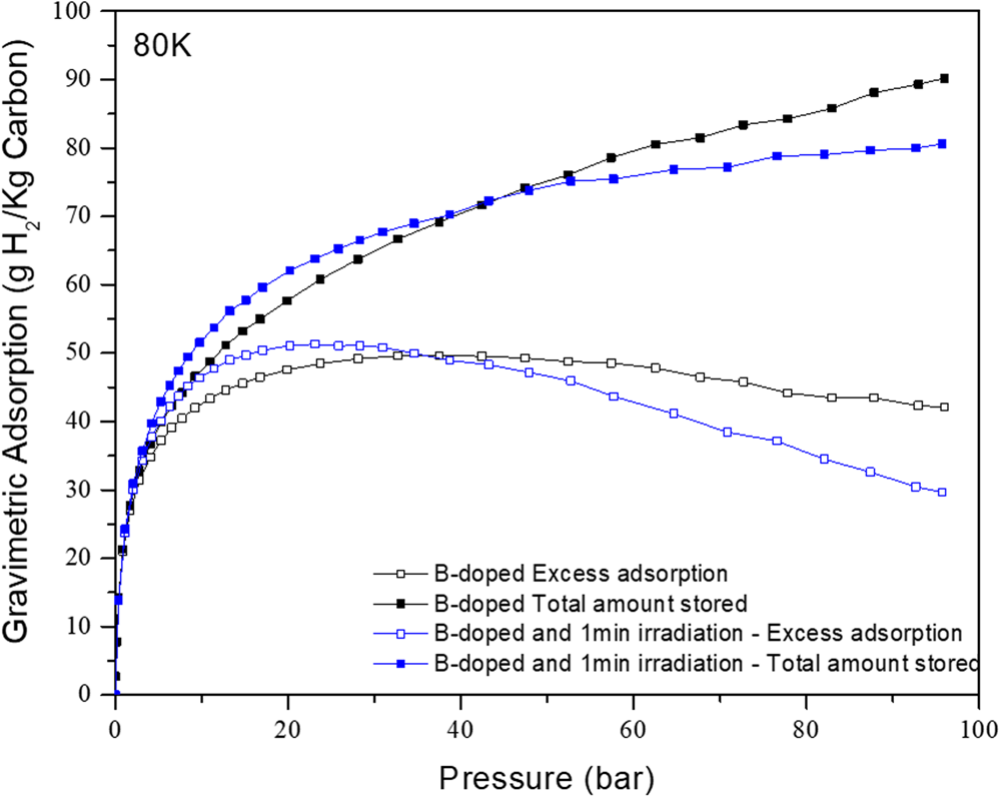
Excess hydrogenadsorption and total amount stored at 80 K for non-irradiated and 1 minute neutron irradiated B-doped samples.

Neutron irradiation of samples for 1 minute caused only a small increase in hydrogen storage at room temperature (compared to the non-irradiated sample). On the contrary, Fig. 4 shows that neutron irradiation modified significantly the shape of the isotherm at 80 K. The pressure at which excess adsorption reached its maximum (23 bar) decreases to about half of the value before irradiation (40 bar). The rapid increase in the excess adsorption at lower pressure is indicative of presence of adsorption sites having higher binding energy. The difference in hydrogen adsorption results from the presence of fission tracks (not easily detectable otherwise).

The lattice gas model developed by Aranovich and Donohue was used to determine the bind- ing energy in the irradiated samples^39,40^. Aranovich and Donohue solved Ono-Kondo equations which relate the density of each adsorbed layer to the bulk gas density and found a general equa- tion for the excess adsorption. Their method was then applied to ACs by Chahine *et al*.^41^, and more recently by Gasem *et al*.^42^. The gravimetric excess adsorption, determined from solving Ono-Kondo equations for slit shaped pores, depends on four parameters: energy of the hydrogen-hydrogen interaction *E*_*H*2*−H*2_ (K), energy of hydrogen-carbon interaction *E* (K), density of the adsorbed film at maximum capacity *ρ*_*mc*_ (g/ml), and a prefactor *C* related to the capacity of the adsorbent for a specific gas. If the gas-gas interaction is neglected, one can reduce the number of parameters to three:

10 $${G}_{e}(P,T)=2C\frac{1-{w}_{1}^{n}}{(1-{w}_{1})(1+{w}_{1}^{n-1})}\frac{1-\frac{{\rho }_{gas}(P,T)}{{\rho }_{mc}}(1-{e}^{\frac{E}{KT}})}{1+(\frac{{\rho }_{mc}}{{\rho }_{gas}(P,T)}-1){e}^{\frac{E}{KT}}}$$

where *G*_*e*_ (*P*, *T*) is the gravimetric excess adsorption, *ρ*_*gas*_(*P*, *T*) is the density of hydrogen at pressure P and temperature T, n is the number of layer in a slit pores of the microporous material and *w*_1_ is a factor which is a function of coordination number, the hydrogen-hydrogen interaction energy, and other variables discussed in detail by Aranovich and Donohue. For n = 2, the excess adsorption can be written as:

11 $${G}_{e}(P,T)=2C\frac{1-\frac{{\rho }_{gas}(P,T)}{{\rho }_{mc}}(1-{e}^{\frac{E}{KT}})}{1+(\frac{{\rho }_{mc}}{{\rho }_{gas}(P,T)}-1){e}^{\frac{E}{KT}}}$$

**Figure 5:**
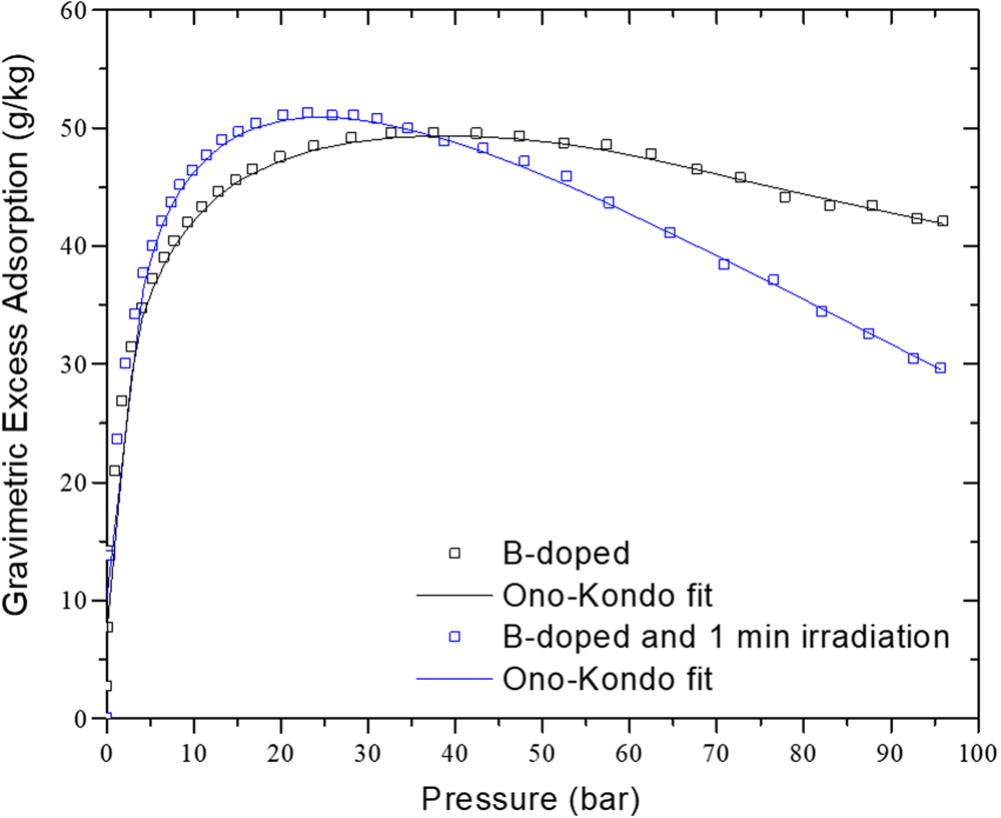
Ono-Kondo fit of gravimetric excess adsorption.

The Ono-Kondo model provides the average binding energy from a single isotherm. Most fre- quently the binding energies are determined from Clausius-Clapeyron equation, using two isotherms at nearby temperatures. This approach is known to be challenging at high coverage because it requires a reliable estimation of the film volume to construct accurate absolute adsorption isotherms^3^. Using the Ono-Kondo model for supercritical excess adsorption^43,44^, the average binding energy can be determined by fitting the experimental excess adsorption in Eq. (11) using Levenberg-Marquardt minimization algorithm. The Ono-Kondo fit for the non-irradiated and irradiated sample is presented in Fig. 5. The estimated average binding energies were KJ/mol and 6.6 KJ/mol for the non-irradiated and irradiated samples, respectively. The 6% increase of binding energy after irradiation is consistent with the hypothesis that boron-neutron capture creates fission tracks, in the form of ultra-narrow pores and surface defects, that adsorb hydrogen with high binding energies. In fact, the defects introduced by the fission tracks (edge defects, free radicals, etc…) serve as sites of higher binding for hydrogen and provide higher stor- age capacities at pressures below 42 bar. For instance, at 20 bar the irradiated sample showed an improvement of 9% in storage capacities compared to the non-irradiated sample. This in- crease in hydrogen storage capacities at low pressure is due to the increase of binding energy at low coverage. While the hydrogen storage capacities of the irradiated material are still below the DOE target, this moderate increase in binding energy provides larger hydrogen storage capacities in the low pressure range which is crucial in assessing the tank deliverable metrics for practical low-pressure applications.

## Conclusion

We showed that neutron irradiation of boron-doped activated carbons alters the surface and pore structure, introducing defects that act as high energy binding sites for adsorbed hydrogen. It leads to a 6% increase of average binding energies in irradiated samples with respect to their non- irradiated parent samples. In addition, this increase is larger at low coverage, resulting in an increase of 9% in the hydrogen storage capacities in the low pressure range (p < 42 bar). The defects introduced by fission tracks cannot be probed using sub-critical nitrogen adsorption, as their diameters are much smaller than those of N_2_ molecules.

## Data Availability

All data generated or analyzed during this study are included in the article. Raw hydrogen and nitrogen adsorption isotherms are available from the corresponding author upon request.

## References

[1] Züttel A (2003). Materials for hydrogen storage. Materials today.

[2] Sakintuna B, Lamari-Darkrim F, Hirscher M (2007). Metal hydride materials for solid hydrogen storage: a review. International journal of hydrogen energy.

[3] Firlej L, Beckner M, Romanos J, Pfeifer P, Kuchta B (2014). Different Approach to Estimation of Hydrogen-Binding Energy in Nanospace-Engineered Activated Carbons. The Journal of Physical Chemistry C.

[4] Romanos J (2017). Cycling and Regeneration of Adsorbed Natural Gas in Microporous Mate- rials. Energy & Fuels.

[5] Rash T (2017). Microporous carbon monolith synthesis and production for methane storage. Fuel.

[6] Romanos J (2016). High surface area carbon and process for its production. US Patent.

[7] Kuchta B (2013). Open carbon frameworks - a search for optimal geometry for hydrogen storage. Journal of Molecular Modeling.

[8] Romanos J, Barakat F, Dargham SA (2018). Nanoporous Graphene Monolith for Hydrogen Storage. Materials Today: Proceedings.

[9] DOE. Technical Targets for Onboard Hydrogen Storage for Light-Duty Vehicles, https://www.energy.gov/eere/fuelcells/doe-technical-targets-onboard-hydrogen-storage-light-duty-vehicles (2017).

[10] Chae HK (2004). A route to high surface area, porosity and inclusion of large molecules in crystals. Nature.

[11] Kuchta B (2012). Hypothetical high-surface-area carbons with exceptional hydrogen storage capacities: open carbon frameworks. Journal of the American Chemical Society.

[12] Romanos J (2013). Infrared study of boron–carbon chemical bonds in boron-doped activated carbon. Carbon.

[13] Romanos J (2012). Nanospace engineering of KOH activated carbon. Nanotechnology.

[14] Jordá-Beneyto M, Suárez-García F, Lozano-Castelló D, Cazorla-Amorós D, Linares- Solano A (2007). Hydrogen storage on chemically activated carbons and carbon nanomaterials at high pressures. Carbon.

[15] Reyhani A (2011). Hydrogen storage in decorated multiwalled carbon nanotubes by Ca, Co, Fe, Ni, and Pd nanoparticles under ambient conditions. The Journal of Physical Chemistry C.

[16] Yang RT (2000). Hydrogen storage by alkali-doped carbon nanotubes-revisited. Carbon.

[17] Kuchta B, Firlej L, Pfeifer P, Wexler C (2010). Numerical estimation of hydrogen storage limits in carbon-based nanospaces. Carbon.

[18] Spalaris CN, Bupp LP, Gilbert EC (1957). Surface Properties of Irradiated Graphite. The Journal of Physical Chemistry.

[19] Thrower, P. A. Impurity nucleation of irradiation damage in graphite. *Journal of Nuclear Materials* **12**, 56–60 (1964).

[20] Cadenhead DA (1964). Neutron irradiation and surface homogeneity of graphitic materials. Carbon.

[21] Chung T, Chung CC (1999). Increase in the amount of adsorption on modified activated carbon by using neutron flux irradiation. Chemical Engineering Science.

[22] Chung TW, Chung CC (1998). Increase in the amount of adsorption on modified silica gel by using neutron flux irradiation. Chemical Engineering Science.

[23] Diduszko R, Swiatkowski A, Trznadel BJ (2000). On surface of micropores and fractal di- mension of activated carbon determined on the basis of adsorption and SAXS investigations. Carbon.

[24] Setoyama N, Ruike M, Kasu T, Suzuki T, Kaneko K (1993). Surface characterization of microporous solids with. Langmuir.

[25] Harris, P. J. F., Liu, Z. & Suenaga, K. Imaging the atomic structure of activatedcarbon, *Journal of Physics Condensed Matter* **20** (2008).

[26] Romanos J (2014). Engineered porous carbon for high volumetric methane storage. Adsorption Science & Technology.

[27] Py X, Guillot A, Cagnon B (2004). Nanomorphology of activated carbon porosity: Geometrical models confronted to experimental facts. Carbon.

[28] Yang S, Hu H, Chen G (2002). Preparation of carbon adsorbents with high surface area and a model for calculating surface area. Carbon.

[29] Romanos J, Dargham SA, Roukos R, Pfeifer P (2018). Local Pressure of Supercritical Ad- sorbed Hydrogen in Nanopores. Materials.

[30] Kaneko K, Ishii C, Ruike M, Kuwabara H (1992). Origin of superhigh surface area and micro- crystalline graphitic structures of activated carbons. Carbon.

[31] Leofanti G, Padovan M, Tozzola G, Venturelli B (1998). Surface area and pore texture of catalysts. Catalysis Today.

[32] Lastoskie C, Gubbins KE, Quirke N (1993). Pore size distribution analysis of microporous carbons: a density functional theory approach. The journal of physical chemistry.

[33] Jagiello J, Olivier JP (2013). 2D-NLDFT adsorption models for carbon slit-shaped pores with surface energetical heterogeneity and geometrical corrugation. Carbon.

[34] Jagiello J, Thommes M (2004). Comparison of DFT characterization methods based on N2, Ar, CO2, and H2 adsorption applied to carbons with various pore size distributions. Carbon.

[35] Gennady YG, Matthias T, Katie AC, Alexander VN (2012). Quenched solid density func- tional theory method for characterization of mesoporous carbons by nitrogen adsorption. Carbon.

[36] Alexander VN, Yangzheng L, Peter IR, Matthias T (2009). Quenched solid density functional theory and pore size analysis of micro-mesoporous carbons. Carbon.

[37] Lide, D. R. *CRC handbook of chemistry and physics on CD-ROM* (CRC Press, 2005).

[38] Steele, W. A. *The interaction of gases with solid surfaces* (Pergamon Press, Oxford, New York, 1974).

[39] Aranovich GL, Donohue MD (1995). Adsorption isotherms for microporous adsorbents. Car- bon.

[40] Aranovich G, Donohue MD (1996). Adsorption of Supercritical Fluids. Journal of Colloid and Interface Science.

[41] Bénard P, Chahine R (1997). Modeling of High-Pressure Adsorption Isotherms above the Critical Temperature on Microporous Adsorbents: Application to Methane. Langmuir.

[42] Sudibandriyo M, Mohammad SA, Robinson JRL, Gasem KAM (2010). Ono-Kondo lat- tice model for high-pressure adsorption, Fluid Phase. Fluid Phase Equilibria.

[43] Sudibandriyo M, Mohammad SA, Robinson RL, Gasem KAM (2011). Ono–Kondo Model for High-Pressure Mixed-Gas Adsorption on Activated Carbons and Coals. Energy & Fuels.

[44] Bi H (2017). Ono–Kondo Model for Supercritical Shale Gas Storage: A Case Study of Silurian Longmaxi Shale in Southeast Chongqing, China. Energy & Fuels.

